# An assessment of priorities in handling climate change impacts on infrastructures

**DOI:** 10.1038/s41598-024-64606-3

**Published:** 2024-06-19

**Authors:** Walter Leal Filho, Roberto Ariel Abeldaño Zuñiga, Javier Sierra, Maria Alzira Pimenta Dinis, Laura Corazza, Gustavo J. Nagy, Yusuf A. Aina

**Affiliations:** 1 Research and Transfer Centre “Sustainable Development and Climate Change Management”, Hamburg University of Applied Sciences, Ulmenliet 20, 21033 Hamburg, Germany; 2 Department of Natural Sciences, Manchester Metropolitan University, Chester Street, Manchester, M1 5GD UK; 3 Centre for Social Data Science. Faculty of Social Sciences, University of Helsinki, Helsinki, Finland; 4 PostGraduate Department, University of Sierra Sur., Oaxaca, Mexico; 5 Department of Applied Economics, Research Center on Global Governance (CIGG), Faculty of Law, Educational University Research Institute (IUCE), University of Salamanca, Paseo Tomáds y Valiente, Salamanca, Spain; 6 Fernando Pessoa Research, Innovation and Development Institute (FP-I3ID), University Fernando Pessoa (UFP), Praça 9 de Abril 349, 4249-004 Porto, Portugal; 7 Marine and Environmental Sciences Centre (MARE), University of Coimbra, Edifício do Patronato, Rua da Matemática, 49, 3004-517 Coimbra, Portugal; 8 Department of Management, University of Turin, Turin, Italy; 9 Instituto de Ecología y Ciencias Ambientales (IECA), Posgrado en Ciencias Ambientales, Facultad de Ciencias, Universidad de la República (UdelaR), Iguá 4225, 11400 Montevideo, Uruguay; 10 Department of Geomatics Engineering Technology, Yanbu Industrial College, 41912 Yanbu, Saudi Arabia; 11 European School of Sustainability Science and Research, Hamburg, Germany

**Keywords:** Climate change, Infrastructure, Transport, Settlements, Floods, Early warning systems, Environmental sciences, Environmental social sciences

## Abstract

Climate change (CC) will likely significantly impact the world’s infrastructure significantly. Rising temperatures, increased precipitation, and rising sea levels are all likely to stress critical infrastructures (CI). Rising temperatures can lead to infrastructure damage from extreme heat events. This can cause roads and bridges to buckle or crack, leading to costly repairs and potential traffic disruptions. In addition, heat waves can damage vital electrical infrastructure, leading to widespread power outages. In light of this context, this article reports on a study which examined the connections and impacts of CC on infrastructure. The study employed a mixed-method approach, combining bibliometric analysis for the period 1997–2022 with a series of relevant case studies from the five continents to offer insight into the impact of CC on infrastructure. The article fills a research gap in respect of assessments of the extent to which climate change (CC) negative influences the infrastructure, with a special focus on developing countries. It also showcases CI projects and adaptation measures being currently deployed, to address CC. The results show that the current infrastructure is vulnerable to CC. The selected case studies on CI adaptation show that in developing and industrialised countries, there is a perceived need to understand better the connections and potential impacts of CC on critical areas such as transport, settlements, and coastal infrastructure. In order to protect infrastructure from CC impacts, governments need to invest in measures such as flood control, early warning systems, and improved building codes. Additionally, they need to work to reduce greenhouse gas emissions more actively, which are the primary cause of CC.

## Introduction

A future imperative for new global infrastructures will be represented by low Greenhouse Gases (GHG) transitions (strategies and actions aimed at reducing the emissions of greenhouse gases, particularly carbon dioxide) to mitigate climate change CC), which involve shifting our energy production away from sources that release significant amounts of GHGs, such as fossil fuels, toward cleaner alternatives that emit little to no GHGs, while also being resilient to the effects of CC. Because infrastructure assets have a long lifespan, decisions made now will lock in vulnerability if these impacts are not already considered during the design phase. The scale of investment decisions is significant. The Organization for Economic Cooperation and Development (OECD) estimated that USD 6.3 trillion per year in infrastructure investment will be required globally between 2016 and 2030 to keep up with development^1^.

These numbers do not include the real financial impact due to mitigation and adaptation investments needed for existing infrastructures. Keeping this in mind, this paper aims to contribute to the recent literature on the existing nexus between adapting infrastructures to CC and the managerial implication due to climate-related hazards. Specifically, this paper is focused on a sub-category of infrastructures that represents a vital role in socio-economic systems, namely critical infrastructures (CI).

The European Commission^2^ defines CI as a “point, system, or part of one […] essential for maintaining the vital functions of a society, and the health, safety, security and economic and social well-being of the community, whose cessation or destruction would have a significant impact". CI forms the basis for many sectors, including transport, energy, communication, water supply, health, and finance^3^.

A CI is created when different systems, people, and ecological contexts are integrated. Therefore, it can refer to an asset, a system, or a part of it necessary to ensure society's basic functioning. Such systems and integration must be planned and designed to ensure resilience and little disturbance, as an improper function will affect society^4^.

A key example of CI is supplying vital energy services, including electricity, gas, and heat, to the global population. Disruptions in such vital services trigger a series of events that result in a dysfunctional society, as seen in the dwindling infrastructure of public health systems and emergency services that were crippled by the effects of COVID-19^4^.

Well-designed CI is a crucial contributor to national prosperity. It is a necessary precursor to economic growth and expansion. Improving productivity in different countries further enhances the quality of life. It leads to economic progression by driving growth, job creation and efficiency. It forms the basis of growth by providing the support networks necessary for processes. Investment in CI allows for the proper functioning of the economy and the growth of businesses through efficiency and connectivity^5^.

The United States of America has identified 16 CI sectors that are imperative to the functioning of society. These sectors include chemical facilities, commercial facilities, dams, communications, critical manufacturing, industrial defence bases, emergency services, energy, financial services, food/agriculture, government facilities, healthcare, nuclear power, information technology, transportation, and water and waste management systems. The incapacitation or destruction of any of these sectors would debilitate the security, national economic security, national public health or safety, or a combination of several cascading consequences^6^.

Table 1 displays the primary characteristics of critical infrastructure across ten chosen sectors.

**Table 1:** Main features of critical infrastructure in ten selected sectors.

Sector	Feature	References
Banking and finance	Provision of monetary means, transactions, and support to economic activities	CISAgov^7^
Government services	Implementation of legislation and governance systems	Westermeier^8^
Information and Communication technologies (ICT)	Provision of communication services	Faisal^9^
Emergency services	Handling accidents, civil protection, and rescue services	Koski^10^
Energy sector	Provision of power/electricity	Bogdanov^11^
Health sector	Provision of medical services and health care	Gupta^12^
Transport sector	Movement of passengers and goods, handling of logistics and supply chains	Combs^13^
Water sector	Management of water supplies and distribution	Tian^14^
Agriculture sector	Food production and distribution	Pawlak^15^
Environmental sector	Conservation of natural resources and protection of ecosystems	Springer^16^

CIs are tangible and intangible systems and assets that provide a country with an essential service whose disruption could generate security, economic stability, public health, safety, or various concatenated problems.

This study departs from two research questions:

1. What is the extent to which different types of infrastructure have been tackled in the literature in association with CC?
2. What are the lessons which can be learned from case studies, on how to prevent impacts of CC on infrastructure?

The authors conducted a mixed-methods study (literature review and case studies) to address these questions. This study helped shed light on some of the issues associated with the topic and is discussed in the methods section of this article.

### Effects of climate change on critical infrastructure

Although this concept originated in the 1990s in a US context, CI is essential in every continent. Therefore, a specific European legislative proposal is also designed to invite individual member states to identify those representing a material level of criticality in maintaining vital societal functions^2^. The notion of criticality is thus related to the possibility that temporary or permanent damage may stop the operation of the infrastructure. In this sense, criticality is seen in vulnerability^17^, which is thus understood to be the relationship between two elements related to the notion of risk: (i) The predictability of a given risk occurring, and (ii) the severity of the associated consequences, which could impact the functioning of the CIs.

Regarding the predictability perspective, there are different types of risks, from those that are technical and human-dependent, such as terrorist attacks^18^, to risks that are more related to socio-environmental systems and natural and meteorological phenomena, as in the case of earthquakes and tidal waves^19^. Both categories represent different degrees of predictability. While regarding the associated consequences, they could be analysed from a short-term or long-term perspective. Direct impact issues, i.e., when the infrastructure is damaged, may result in the temporary or prolonged suspension of the service offered, and permanent and irreversible damage results in complete disruption that may persist over time. In addition, a further difference exists between direct and indirect impacts, i.e., the series of malfunctions and disruptions that cascade to other related CIs^20^.

Further peculiar aspects of these infrastructures are also represented by the mutual interconnection between different infrastructures and the increasingly real possibility that the same CI can connect and impact the functioning of two or more states. In the former case, the disruption of service of one CI, a sort of node^21^, could directly impact the operation of other CIs connected to it through a cascading effect^20^. An example of this is the disruption of electrical services, which could slow the operation of hospitals in a specific area. In the second case, reference is made to interconnections between different states; therefore, the CI represents a unifying element^22^. Think of a highway, tunnel, bridge, or rail line; in this case, interconnections between different states would also be disrupted so that the resulting impacts would go beyond the geographic boundaries of a specific nation.

Research in CI and impacts resulting from CC is still embryonic. However, its relevance to global economies is specific, as global infrastructure is essential for achieving the SDGs. In this sense, the predictability of CC impacts through adopting adaptation and mitigation strategies is crucial^19^. For example, rising sea levels or coastal erosion is a highly likely risk for all those CIs that find their geographic location in coastal areas (think nuclear power plants, ports, and oil/gas pipelines)^23^. In addition, other impacts resulting from CC may also affect the vulnerability of CI, such as heatwaves, extreme weather events, floods, and storms^19^.

Precisely, the vulnerability of a CI to impacts from CC may imply a different temporality before direct consequences on the operation and degradation of the infrastructure itself can be seen^24^. Next to instantaneous events, where the rapidity of such an event is high, there are also slower and more protracted effects over time, which can result in permanent operation disruption if not adequately monitored^25^ (Table 2). As a result, it is clear that at the political and managerial level, the issue of CIs is addressed in terms of preservation policies aimed at increasing the robustness of a specific infrastructure, ensuring an improvement in its reliability and resilience^26^. Therefore, the researchers contemplate the possibility of an interruption of a CI as admissible. However, resilience indicates the ability of the CI itself to recover quickly and to return to function at the same level of ante-disruption by absorbing the generating causes, ensuring that the risk of interruption decreases (prevention) over time and that the recovery time between one event and the next one progressively reduces (absorb, adapt, recover) in a predetermined way^27^. Therefore, CI resilience goes beyond CI protection, implying a proper level of equipment in terms of robustness, redundancy, resourcefulness, and rapidity^28^.

**Table 2:** Effects of climate change on infrastructure.

Infrastructure	Climate Change Impact	References
Roads	Damages by floods or by extreme heat, which melts the asphalt and degrades roads	Amin^29^
Bridges	Bridges may be damaged or become unstable due to floods or gale-force winds during cyclones	Argyroudis^25^
Water supplies	Droughts may result in the depletion of water supplies	Pokhrel^30^
Waste Management Systems	Increased temperatures may accelerate waste degradation and release more greenhouse gases	Wang^31^
Irrigation	Diminished water supplies may result in the need for reduced irrigation as a water conservation strategy	Sánchez-Blanco^32^
Power generation	Extreme events may damage power links Production of hydropower may be curbed due to the limited availability of water in the summer months	Thacker^33^

A further aspect of interest, which unites CIs with megaprojects, is that the governance and financing of these infrastructures are often only sometimes linear. However, they could be hybrid or fragmented^28^. There may be different types of CI governance, from public to entirely private or to forms of public–private collaboration. Alternatively, some infrastructures can be managed by different countries and their respective governments. Consequently, the governance of a CI is an essential feature for the social and political implications that derive from it to increase the systems’ resilience^26^. Sometimes the decision-making process goes beyond a single state or institution but can connect business networks or intergovernmental agreements. Financial and insurance aspects also become critical elements of CIs. In particular, a CI has likely taken a long time to build and enter into operation. Moreover, the funding for its operation may concern different subjects, including supranational institutions such as the European Union or the World Bank^34^.

Therefore, decisions regarding the impacts of CC on CIs may require complex processes and need to be more easily implemented^35^. Therefore, it implies that at a global level, the topic of climate-resilient infrastructure is a theme of absolute importance because it may require long negotiation and implementation processes at a national and supra-national level to upsurge.

## Materials and methods

The research on CI and the impacts of CC on it is still in its early stages. In this context, the literature reveals a significant gap in understanding successful adaptations of CI to cope with climate-related hazards, a topic that our research aims to address in a novel and comprehensive way.

The literature analysis aimed to explore the impacts of CC on infrastructure across various contexts, such as human settlements, transportation, and coastal areas, among others, rather than focusing solely on a single sector, as is often the case in many studies. Additionally, it sought to identify the extent of coverage of the topic in peer-reviewed work.

To answer the first research question, “What is the extent to which different types of infrastructure have been addressed in the literature in association with CC?” our research team undertook a rigorous qualitative study^36^ that was not a mere exploration but a comprehensive effort. We aimed to understand the impacts of CC on infrastructure across various contexts, such as human settlements, transportation, and coastal areas, departing from the norm of focusing solely on a single sector. This approach allowed us to gauge the extent of coverage of the topic in peer-reviewed works, a crucial aspect in the field of CC and infrastructure.

The literature review was conducted using the Scopus database as the primary source. Scopus is a comprehensive multidisciplinary abstract and citation database that covers a wide range of scientific disciplines, including those relevant to the study of CC and infrastructure. Its vast array of peer-reviewed journals, conference proceedings, and other scholarly literature gives researchers the confidence to conduct comprehensive searches and access relevant publications from various fields, setting it apart from more restricted databases.

The literature search combined the following keywords: ‘Critical Infrastructure', ‘Climate change', and ‘adaptation', in the Scopus database^37^. between 1997 and 2022. Two researchers in the team cross-validated this search strategy, ensuring the reliability of the results. The initial search yielded 270 articles, subjected to a rigorous selection process.

Several inclusion and exclusion criteria were meticulously applied to identify research articles in English published in scientific journals. Consistent with previous studies^38^, two research team members reviewed all articles' titles and abstracts and removed 120 articles unrelated to the topic or those not published as original research. In case of disagreements, a third researcher assessed the article before making the final decision about inclusion or exclusion. The articles were not filtered by year of publication, ensuring a comprehensive and unbiased selection process.

**Figure 1:**
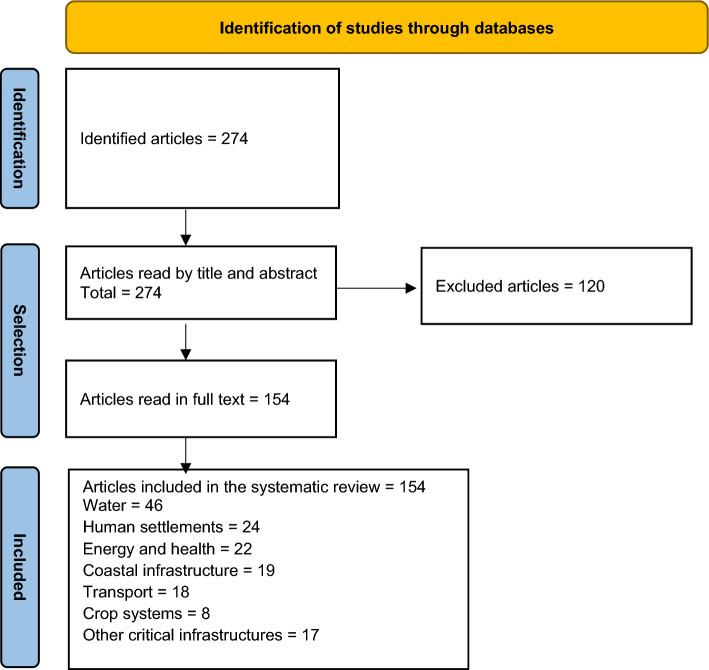
Flow diagram of included studies.

At the end of this process, 154 articles were classified into the following categories: water (*n* = 46 articles), human settlements (*n* = 24), coastal infrastructure (*n* = 19), transport (*n* = 18), crop systems (*n* = 8), energy and health (*n* = 22), and other CI (*n *= 17) (Fig. 1). The subsequent section analysed and summarised these articles in detail.

In the study's second phase, the research team addressed the second research question, "What lessons can be learned from case studies on how to prevent impacts of CC on infrastructure?" To this end, the authors complemented the literature review with a search of reports and case studies on Google. Google, the most popular search engine globally, was chosen due to its widespread usage. Thus, critical case studies of CI projects and adaptation measures to address CC were identified and analysed.

The criteria for selecting the case studies are threefold. First, there was a need to ensure a representation of various geographical regions. Second, there was a need to represent different sectors and avoid focusing on one only since CC impacts on infrastructure are widely spread. The final criterion used in selecting case studies was identifying and documenting experiences that may be replicable elsewhere.

The authors selected the final set of articles and case studies to show a representative picture of successful projects implemented in low-income, middle-income, and upper-middle-income countries. According to Yin's^39^ , each case study considered a type I was assessed separately.

Issues addressed in the selection of these case studies include the following ones:

- *Geographic Scope—*Ensuring representation from diverse regions, capturing a wide range of climate-related hazards and adaptation strategies.
- *Sectoral Coverage—*Including CI sectors such as coastal infrastructure, transportation, flood risk management, settlements, crop systems, energy, and water infrastructure to provide a comprehensive overview.
- *Temporal Considerations*:—Spanning different time periods to capture evolving climate challenges and adaptation responses.
- *Impact Assessment—*Assessing the effectiveness and implications of adaptation strategies, including economic, social, and environmental aspects.
- *Policy and Governance—*Examining the role of policies, regulations, and governance structures in facilitating or hindering adaptation efforts.
- *Interdisciplinary Approach—*Integrating insights from disciplines such as engineering, urban planning, agriculture, and policy studies to offer holistic perspectives on adaptation challenges and solutions.
- *Stakeholder Engagement—*Involving key stakeholders, including government agencies, local communities, and private sector actors, to ensure relevance and applicability of adaptation measures.
- *Transferability of Lessons—*Identifying lessons learned and best practices that can be replicated or adapted in other contexts facing similar climate risks.

The following section presents the results in detail.

Figure 1 schematises the flow diagram of included studies.

## Results and discussion

This section presents the results of the analysis of the impacts of CC on different sectors and human activities. The first part had an exploratory approach and highlights the trend in the publication of CC impacts on infrastructure and the nature of the adaptation projects. The second part had an explanatory approach. It discusses the impacts of CC on infrastructure in different sectors and the adaptation strategies being implemented to address the impacts, and presents the lessons learned from the selected case studies of eight CI projects across the globe.

### Bibliometric analysis

**Figure 2:**
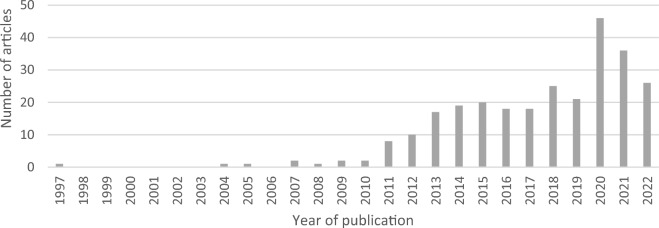
Articles included in the review by year of publication between 1997 and 2022. Source: Literature search on Scopus.

The initial search in Scopus using the combination of the terms ‘Critical Infrastructure’, ‘Climate Change’ and Adaptation, without time restrictions, yielded 274 research articles after excluding books, conference papers, and other documents. The main volume of articles that address this issue was published from 2018 onwards (154 papers) (Fig. 2).

**Figure 3:**
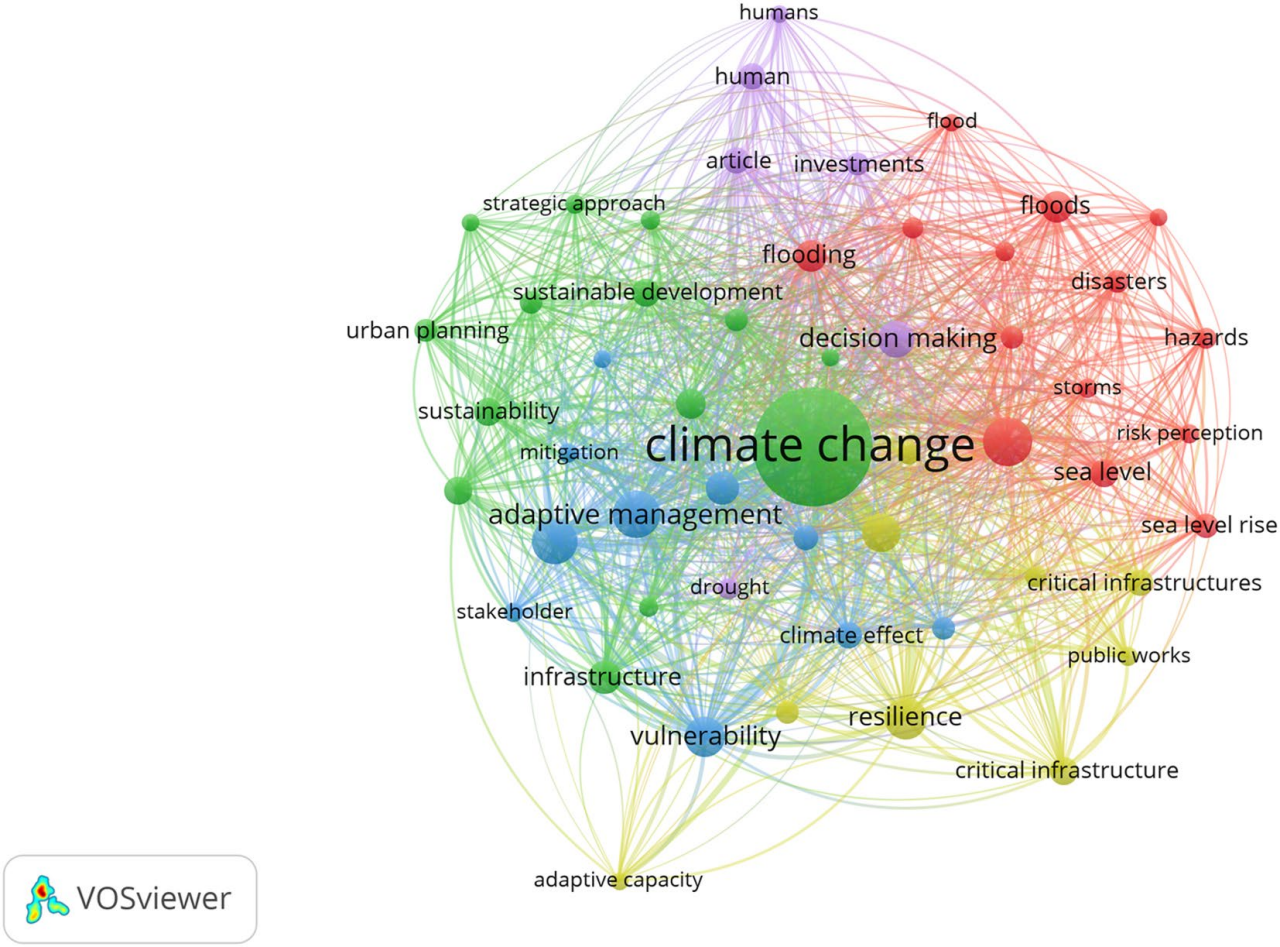
Network map of keywords included in the reviewed articles. Source: Literature search on Scopus.

The authors analysed the keywords contained in the reviewed articles and listed those keywords with a minimum of 20 occurrences to draw a keywords network map, aiming to retrieve thematic (words) clusters within the literature. We identified five clusters shown in Fig. 3. Each colour represents a cluster. The first cluster, represented in red, contains the 14 keywords related to the hazards, and the most frequent keywords were: disasters, flood, climate models, hazards, sea level, and storms, among others. The second cluster, represented in green, contains 14 keywords related to planning and decision-making issues, with CC, infrastructure, urban planning, sustainable development as the most frequent keywords, among others. The third cluster, represented in blue, included 10 keywords related to adaptive management, vulnerability, stakeholder, and mitigation as the most frequent words. The fourth cluster, represented in yellow, included nine keywords related to CI, public works, resilience, and adaptive capacity. Finally, the fifth cluster, represented in purple, included six keywords related to humans, investments, and articles as the most frequent words.

The study included the review of the 154 articles retrieved from the last five years. The articles refer to studies conducted in countries of the five continents. The sectors included in the studies were airports (1 article), coastal infrastructure (14 articles), CI (as a whole) (15 articles), crop systems (8 articles), dams (1 article), digital technology (2 articles), economy (1 article), energy (5 articles), food supply (1 article), health (5 articles), landscapes (3 articles), mining (1 article), oil industry (2 articles), roads (5 articles), seaports (5 articles), settlements (24 articles), touristic infrastructure (1 article), transport (13 articles), waste (2 articles), and water (Flood risk, irrigation, and resources) (46 articles). The main research articles are focused on infrastructure related to water, either as a resource or as a problem within coastal infrastructures and human settlements, which confirms the results showed in the previous cluster analysis.

Concerning the infrastructure adaptation proposals reviewed, 27 articles refer to implemented and currently operating adaptation projects. In comparison, 127 articles present proposals for integrative approaches and mathematical models considering climate projections for potential future adaptations. These results show that research focuses on modelling and projections to estimate the future impacts of CC, with a clear emphasis on integrative approaches to deal with its negative consequences.

#### Climate change and water infrastructure

Consistent with the relevance that CC and its related effects on water infrastructure have in the literature^40^, 27 articles within the scope of the effects of CC on water infrastructure focus on evaluating flood risk, representing the most extensive set of documents within this review. Some authors use modelling approaches to improve the estimation of the timing^41^ and the dimension of alterations in terms of discharges, water levels, and flood dynamics, which may determine future development scenarios^42^, confirming that severe floods produce the most significant erosion and deposition events and that the likelihood for this phenomenon will be more extensive in the future^40^.

Other studies have addressed the issue of urban planning and adaptation to deal with the challenges generated by pluvial floods in cities for critical public services^43^, where differences in infrastructures between urban areas may be relevant for vulnerability and resilience^44^. These results align with the threats that severe floods pose for urban areas, where CI may be damaged and the provision of essential services, which are crucial for the normal functioning of society, could be at risk^45^. To address this issue and taking into consideration these potential risks, some studies suggest counterbalancing measures, such as 'thickening' soft regional structures and relocating density to less vulnerable zones^46^, designing alternative sanitation interventions for flood scenarios, and managing the consequences that floods may generate in highly polluted areas^47^.

In this context, some articles have explored system dynamics and decision-making processes^48^, aiming to assess perceptions of CC and adaptation needs by public authorities^49^. However, it is well known that modelling and implementing resilience to urban flooding is challenging and complex^50^. Therefore, these results confirm that the participation of different stakeholders is essential for effective flood risk management, as well as developing and implementing participatory methods for integrating CC adaptation into community planning^51^.

Some studies explore risks for water as a resource from diverse perspectives. Within this sphere, challenges for water provision are the main relevant issue, either at the city level in normal circumstances^24^, in rural areas^52^, or at specific locations inside cities where different events led to high concentrations of people in specific points on time^53^. These studies suggest that interactions, risks, and potential synergies between different water-intense sectors are crucial to fully understanding and improving water management^54^. Different structural and non-structural^55^ approaches to improving water use could be implemented to address these problems. The difference between structural and non-structural measures relies on the fact that the former requires significant investments and generates lasting, sometimes permanent changes. At the same time, the latter presents the advantage of guaranteeing access to water in acute episodes while eluding significant investments, which could end up underused in favourable climate circumstances^56^. In line with the abovementioned need to enhance the participation of different stakeholders and promote citizen participation, various studies have also analysed this issue from the governance perspective, either focusing on improving management, monitoring, and supervision by authorities or aiming to improve water management by promoting citizen participation and engagement^57^.

In addition, six articles included in this review address potential risks generated by CC for irrigation infrastructure, which allows for a rise in the share of water use consumed by crops and alleviates impacts on production. Pérez-Blanco^58^ assessed irrigators' reactions to pecuniary compensations intended to maintain irrigation-dependent ecosystem services and proved that, in general, conservationist approaches have better economic performance and are more robust than autonomous adaptation strategies where modern irrigation systems are implemented. López^59^ followed a similar approach^58^,^59^ that used satellite data on land use and CC in human–environment dynamics to analyse environmental changes and local impacts and adaptations. They suggested that wide-ranging adaptations connected to climate and land use modifications, market features, and complex knowledge schemes are crucial for the resilience of smallholders. In a different context, road water harvesting practices have been used in the drylands of Ethiopia to supplement rain-fed agriculture. This alternative strategy for transformative CC adaptation helped reduce rain-related road damage, erosion, and landscape degradation, while income for farmers increased^60^. Nevertheless, despite the crucial role of irrigation systems in CC adaptation, their implementation is still far from optimal and offers much room for improvement^61^. Besides, irrigation systems may contribute to increasing energy and infrastructure demand and greenhouse gas emissions^62^.

#### Climate change and human settlements

Another area of study that has focused the attention of researchers is the effects of CC on human settlements. In this regard, 24 studies included in this research address this issue from different standpoints. Several articles explore this topic comprehensively^63^, aspiring to identify the multiple challenges CC brings in all spheres of the environment and human life^64^. In this regard, several articles focus on the negative consequences of this phenomenon in cities and urban areas affected by rapid mass urbanisation. It is proven in the literature that this scenario contributes to the heat island effect and reinforces an uneven distribution of precipitation and extreme weather^65^. For this reason, the research aims to explore innovative and sustainable ways of urbanisation^66^, taking into consideration crucial elements such as the vulnerability in urban areas, the higher risks faced by vulnerable populations^67^, marginalisation of disadvantaged groups from green facilities, and stressing the importance to foster community resilience^68^. In this context, research also focuses on assessing resilience strategies, including evacuation decisions^69^ and other planning for emergency scenarios in case of disasters^70^.

In addition to risks for urban areas, several papers have studied the challenges for human settlements in mountain settings and other areas where CC is accelerating and increasing risks for people^71^. Finally, regarding the economic and financial side of CC adaptation, Chirisa^72^ discusses the frameworks required for sustainable climate adaptation finance, a similar topic explored by other authors^73^, who use demographic projections seeking to adopt fiscal and infrastructure planning efforts.

#### Climate change and critical infrastructure

Research on CI for adaptation against CC includes a wide range of tangible and intangible physical, ecological, institutional, and cultural outcomes^74^. In this context, the literature refers to five key issues^75^, namely, disaster theory research from different perspectives, either by focusing on specific contexts^76^ or paying attention to complex networks^33^, the construction of databanks^77^, as well as the estimation of climate hazard projections^35^. In line with this, several studies focused on clarifying fundamental concepts, connections, and causes, aiming to understand better socioeconomic vulnerability and the complex risks associated with CC^78^.

The development of frameworks and models of integrated disaster hazards is another topic addressed by several studies, i.e., to assess urban or suburban water catchment to tackle flood risk^79^, to estimate the required changes in the short, medium and long-term^80^, or to analyse the relevance of social, environmental, and technical elements for infrastructure planning^81^. Another area of research within the sphere of CI focuses on analysing systems and structures for integrated disaster risk evaluation and governance, including multiple connections between disasters, nature, society, and human behaviour. In this regard, some topics addressed by relevant studies are governance structures for improving the integration of the organisation and administration of critical urban infrastructures^82^, interdisciplinary consultation to outline and evaluate decision-oriented processes^83^, or the assessment of the resilience and vulnerability of CI to severe weather events, including fluvial flooding risk^84^.

#### Climate change and coastal infrastructure

Regarding the challenges and risks for coastal infrastructure generated by CC, the sphere that has received the most attention is the analysis of the potential adverse effects of sea level rise on coastal areas and their infrastructure^85^. This scenario may pose risks for the industrial sector and private households^86^. Against this background, several studies provide evidence of the relevance of adaptation measures combining technology and community training and nature-based solutions to increase resilience and reduce vulnerability in coastal areas^87^.

In line with technology, several papers explain how it has been used to design small-scale adaptative proposals^88^ and propose a more comprehensive assessment of the cost-effectiveness of several possible strategies^89^. In this context, technology plays a crucial role in modelling spatiotemporal impacts^90^ and generating digital simulations of flood exposure maps produced using local CC forecasts of sea level rise and storm surge effects^91^. In addition, research also focuses on different adaptation measures^92^, stressing the relevance of nature-based solutions based on elements such as salt marshes or coral reefs^93^. At the same time, several studies addressed the issue of insurance and financing^94^.

Seaports are crucial elements within the scope of coastal infrastructure. In this context, risks and challenges for seaports have been addressed from diverse standpoints, including collaboration among coastal stakeholders^95^, awareness of stakeholders as regards potential risks generated by CC^96^, as well as the barriers that decision makers face to improve adaptation to protect seaports from extreme weather events^97^. Also, some studies focused on assessing projections of forthcoming regulation on current business models or potential variations in international maritime trade under different climate-based settings^98^.

#### Climate change and transport

Numerous studies explore the risks and challenges CC generates on transportation and transport infrastructure. Some articles address potential risks and adaptation strategies for the transportation system^99^, strongly focusing on roads, railways, and bridges^100^. Other studies pay special attention to risks generated by flooding^101^, sea level rise^102^, and avalanches^103^. Some studies also investigate challenges and opportunities for successful adaptation planning and ways to improve decision-making^104^. For this purpose, different models have been used to estimate better alternatives for highway and transit assignment^105^, as well as investment needs and options in the infrastructure’s vulnerable components for safeguarding urban mobility under a potential attack scenario^106^. Special attention has been paid to roads because of the potential risks that heat may cause, stressing the importance of improving pavement management^107^ and guaranteeing access to rural areas, often more exposed to climate-related hazards^108^.

#### Climate change and crop systems

Crop systems are another aspect that may be severely affected by CC. Some studies have explored comprehensive frameworks designed to improve crop management^109^, map climate risks^110^ and evaluate water scarcity risks^111^. Also, issues such as exploring adaptation views^111^, alternatives for diversification of adaptation agricultural activities^112^, and understanding and adjusting farming behaviour via financial means^113^ have been analysed. Against this background, several articles have investigated the potential use of nature-based solutions to improve agriculture production, drought resistance and ecosystem restoration^114^.

#### Climate change and energy generation

The energy sector is another sphere that may be negatively affected by changes generated by CC. Therefore, it is crucial to evaluate the status of energy stability, inequality, and CC vulnerability^115^ and undertake cost-effective assessments required to design and implement feasible adaptation strategies^116^. In addition, current research explores risks for alternative infrastructure and sustainable alternatives based on geothermal heat^117^.

**Figure 4:**
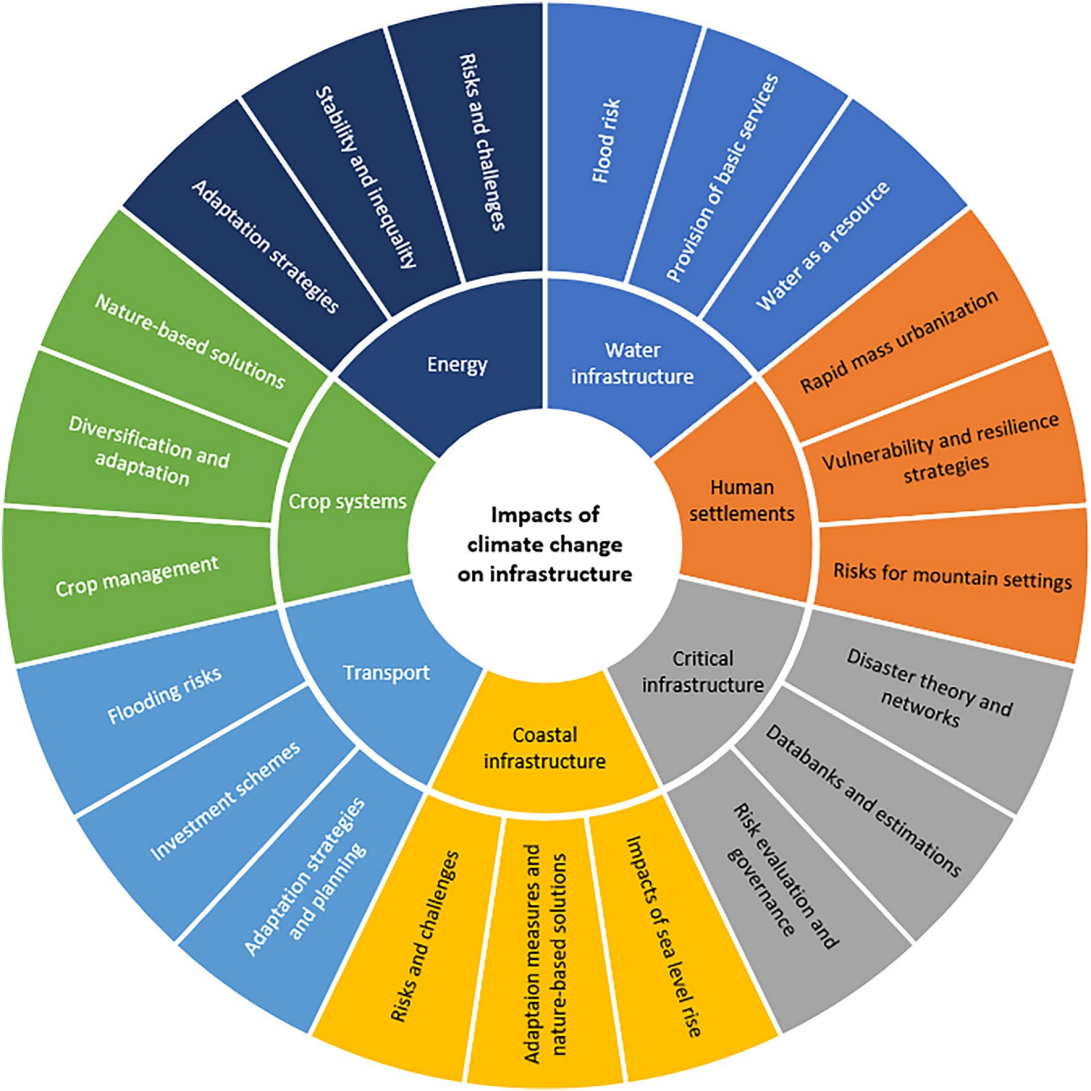
Seven categories and their main issues. Source: elaborated by the authors.

Figure 4 shows the main topics the literature addresses for the seven categories regarding the impacts of CC on infrastructure identified in this research.

### Case studies on specific adaptation measures on critical infrastructures

Eight case studies of CI adaptation projects which suffers from the impacts of CC were selected, representing different regions worldwide. At least one situation per continent was included. The selected cases cover the coastal infrastructure, transport, flood risk, settlements, and energy sectors.

Adapting crucial infrastructure to CC needs to be understood holistically to ensure that communities are not left without essential supplies and services^74^ caused by extreme weather events, requiring adaptation measures to minimise the risks^118^.

The issue of adaptive and resilient CI is becoming more and more relevant as a result of the substantial effects that CC will have on the local-specific^119^ efficiency and viability of CI assets constructed to last for a long time, and thus vulnerable to both fast-onset and slow-onset occurrences^74^. Dense urban ecosystems such as cities have a complex nature. As a result, they are particularly susceptive to CC and extreme weather events, requiring timely responses due to socioeconomic consequences resulting from the vulnerability to CC impacts^118^.

In Africa, the climatological-related scarcity of water, with the possibility of running out of water, affects equitable infrastructural governance priorities associated with CC, contributing to deepening inequity in the region and with implications to the CC adaptation policies^55^. Consistent with this, the Addis Ababa Water and Sewerage Authority, along with the Adama Water Supply and Sewerage Enterprise, undertook a pilot project aimed at enhancing climate-resilient water safety. Since 2014, the implementation of this project involved both drinking water utilities. The pilot initiative demonstrated a strong commitment to policy and national guidance^120^.

CC also can affect energy infrastructure, such as solar and photovoltaics or hydropower systems, through temperature rise, dust, and extreme weather events^118^. At the same time, energy infrastructure will be affected by high energy demand caused by extremely low or high temperatures^121^. In this continent, the Batoka Gorge Hydro Electricity Scheme, which covers Zimbabwe and Zambia, is currently under development in response to water shortages in existing dams that have left large sections of the population without energy in recent years^122^. Interestingly, publications addressing CC impacts on CI in Africa focus on cost and financing^123^ due to the specific problems faced regarding limited funds. That is also why the proposed adaptations need to consider long-term planning^124^ to reconcile competing demands into a coherent CC policy that balances immediate needs and potential future infrastructure implications^125^.

Since roads are exposed to environmental conditions^123^, road infrastructure is one asset that could potentially change due to expected CC effects, e.g., storm surges, flooding, and specific drainage. The significance of taking CC adaptation into account at both the present and future stages of road infrastructure management policy need to be considered, with a proactive road infrastructure adaptation, i.e., dirt roads upgrade, resulting in reduced maintenance costs on non-gravel road use and unpaved roads and reduced sensitivity to the effects of CC^126^. Using pervious surfaces in cities is a way to minimise flooding and contribute to water infiltration^119^. In Japan, the train company's adaptation plan increased the estimated maximum operation temperature for the railways from 60 to 65 °C. In this way they are adapting the transport infrastructure to safely operate in an scenario of increasing temperatures^127^. Transport and road infrastructure is of crucial importance due to extreme weather events, effects of precipitation, temperature, and flooding changes on the paved and unpaved roads, with the price of maintenance and repair being significant, demanding further actions on the associated challenges in terms of policy making^123^. The transportation infrastructure was projected considering historical weather data, now significantly varying and forcing a shift and planned to be resourceful, inclusive, interconnected, solid, and flexible^118^.

In other regions of the world, such as the Middle East^118^, Europe, or the United States^119^, the focus is on projecting models able to assess/model the upcoming extreme weather events effects caused by CC on infrastructure, because of the heavy societal dependence. In specific regions of the world, infrastructure maintenance costs are high, among the most expensive in CC adaptation. Table 3 describes the rationale and essential points of the Eyre Peninsula Plan for adapting coastal infrastructure in Australia, given the increase in coastal flooding in that country. Another case study is the Ultimate Safety Levels project developed by the Japanese train company to adapt its railways to the temperature increase. Regarding Hong Kong, the Sponge City Program was included to deal with flood risks. In the United States, the reconstruction project for New Jersey after the impact of Hurricane Sandy was identified. The “Cloudburst” flood risk management project in Copenhagen is also described.

**Table 3:** Selected case studies on Critical Infrastructure adaptation.

Country	Year	CI/Sector	Rationale	Brief description of the adaptation strategies
Australia	2013	Coastal infrastructure	Increasingly frequent flooding in coastal areas with an impact on coastal infrastructure	The **Eyre Peninsula Plan** outlines the adaptation priorities identified by key regional leaders on the Eyre Peninsula. Through the planning of sequenced pathways, adaptation measures required by individual sectors and needs were identified following these principles: Build economic resilience; prepare not to repair; take joint responsibility; identify long-term decisions; seek out and avoid cross-sectoral maladaptation The priorities of the Plan included an Integrated Management Strategy for Spencer Gulf and Regional Transport Infrastructure Planning^128^
Japan	2019	Transport	Increasing temperatures impact the railways by expanding the steel. It could cause accidents	The train company's adaptation plan increased the estimated maximum operation temperature for the railways from 60 to 65 °C. They have also developed robots to maintain the tracks: “*The operator steers the robot from the cockpit (near the ground), so they can work safely on tasks high in the air*.” In this way, they plan to raise safety by pursuing "**Ultimate Safety Levels**”^127^
Hong Kong	2017	Flood Risk	Hong Kong is in a humid subtropical region with a marine subtropical monsoon climate. Due to its location, it registers a high incidence of tropical typhoons and has one of the highest rainfalls in the Asia Pacific region	The **Sponge City Program** envisions a city's water management system functioning like a sponge (a highly porous structure) to absorb, store, infiltrate, and purify rainwater and release it for reuse when needed This program is implemented by the Drainage Services Department (DSD) of Hong Kong. The nature-based drainage system aims to build flood resilience and improve public spaces^129^
United States	2012	Settlements	In October 2012, Hurricane Sandy made landfall in New Jersey. Hurricane Sandy caused approximately $65 billion in damages and 650,000 damaged or destroyed homes	Immediately after the devastation caused by Hurricane Sandy, President Barack Obama established the **Hurricane Sandy Reconstruction Task Force**. The Task Force coordinated federal interagency efforts to guide a successful Hurricane Sandy rebuilding process The strategy focused on the following points: (1) Aligning funding with local rebuilding visions; (2) Cutting red tape and getting assistance to families, businesses, and communities efficiently and effectively, with maximum accountability; (3) Coordinating the efforts of the Federal, State, and local governments and ensuring a region-wide approach to rebuilding; (4) Ensuring the region is rebuilt in a way that makes it more resilient^130^
Denmark	2012	Flood Risk	In July 2011, in less than two hours, Copenhagen was hit by an extreme one-in-1,000-year storm event ( Cloudburst), where 150 mm of rain left large areas of the city under up to one meter of water. A sizable storm preceded the 2011 event in 2010 and occurred again in 2014. Copenhagen assumed the storms were not a one-time occurrence and projected harbour sea levels to rise by one meter by 2110	The City of Copenhagen developed the **Cloudburst Plan** in 2012, an offshoot of the Copenhagen climate adaptation plan. The Plan describes the priorities and recommended measures for climate adaptation, including extreme precipitation. The City assessed the costs of the different measures (traditional versus different options, including adaptation measures), the cost of damage despite the measures, and the resulting financial impact The implementation of adaptation measures in Copenhagen focused on stormwater drainage to the sea, stormwater storage, and moving flood risk management initiatives into blue-green city infrastructure^131^
Rwanda	2019	Crop systems	Development initiatives can affect smallholder producers’ capabilities to adapt to CC. In this context, ‘green revolution’ agricultural approaches implemented in several countries in sub-Saharan Africa may intersect with cross-scale social-environmental practices to irregularly influence smallholders’ adaptive abilities and adaptation strategies	**Rwanda’s Crop Intensification Program** allows some more prosperous producers to adjust their livelihoods by receiving income from commercial agriculture. Meanwhile, cease of action of local risk management organisations has reduced climate risk management alternatives for most households. Social institutions are crucial for defining diverse adaptation strategies by facilitating or limiting alternatives for smallholders to adjust livelihood and land use approaches. It is necessary to redevelop strategies carefully, considering how power structures and established social disparities can lead to smallholders' uneven abilities to adapt to CC^132^
Zimbabwe and Zambia	2020	Energy	Zimbabwe and Zambia rely on power from the same hydropower assets on the Zambezi River. However, between 2014 and 2019, they suffered drought impacts on hydroelectric power production. As a result, both countries faced extreme energy shortages when reservoir levels dropped to 12% due to low rainfall in 2014 and 2015 and 9% in 2019	**Batoka Gorge Hydro Electricity Scheme** (BHES) is a 2.4 GW hydroelectric project located on the Zambia-Zimbabwe border, on the Zambezi River, approximately 54 km downstream of Victoria Falls. The project is run by the Zambezi River Authority (ZRA), owned by the governments of Zambia and Zimbabwe. The project started in 2020 and is expected to finish in 2026^122^
Ethiopia	2019	Water infrastructure	Several phenomena, such as CC, population growth, rising urbanisation and expanded water demand, are likely to increase water supply pressure. To deal with this scenario, the World Health Organization (WHO) recommends implementing a risk assessment and risk management strategy involving all stages of water supply from catchment to consumer, known as water safety plans (WSP)	The Addis Ababa Water and Sewerage Authority and the Adama Water Supply and Sewerage Enterprise implemented a climate-resilient water safety pilot project. These two utilities are in the Awash basin and use surface water as raw water sources for their supplies. Implementation of the project started in 2014 in both drinking water utilities. This pilot project showed policy commitment and national guidance. In addition, it highlighted that strategies to deal with reduced water quantity availability might not only include the development of other water sources but can also incorporate optimising water demand management in a water supply, adjusting water prices, refining domestic water use technology efficiency and water harvesting, and dropping non-revenue water^120^

The experiences from the case studies are summarised in Table 3.

The case studies addressed in Table 3 provide several valuable insights for CI adaptation to CC, highlighting the critical imperative of comprehensive planning, innovation, collaborative governance, social equity, diversification of energy sources, and integrated risk management in fortifying climate-resilient CI systems. Demonstrated by initiatives such as the Eyre Peninsula Plan in Australia and the Cloudburst Plan in Denmark, comprehensive adaptation plans spanning multiple sectors and considering long-term climate-related hazards are pivotal^127,130^. Moreover, the utilization of innovative technologies like robots for railway maintenance in Japan and nature-based drainage systems in Hong Kong bolsters infrastructure resilience and safety^126,128^. Collaborative governance, exemplified by the Hurricane Sandy Reconstruction Task Force in the United States^130^, and the Sponge City Program in Hong Kong^129^, underscores the significance of coordinated efforts among government agencies, local communities, and stakeholders. Addressing social disparities and ensuring equitable access to adaptation measures, as seen in the Crop Intensification Program in Rwanda^132^, and the development of climate-resilient water safety plans in Ethiopia^120^, is crucial for fostering resilience. Furthermore, the diversification of energy sources, showcased by projects like the Batoka Gorge Hydro Electricity Scheme in Zimbabwe and Zambia, mitigates the impacts of climate variability on energy production^122^. Integrated risk management strategies, such as water safety plans in Ethiopia, are essential for addressing the multifaceted challenges posed by CC and other socio-economic factors affecting water infrastructure^120^. Overall, the presented case studies underscore the importance of proactive planning, innovation, collaboration, and social equity in building climate-resilient CI systems.

## Conclusions

Infrastructure is vital for modern societies, providing essential services and driving economic growth. However, CC poses significant challenges to infrastructure resilience and sustainability globally. While there is growing focus on how CC affects infrastructure in industrialised nations, research on its impact on developing countries' infrastructure is lacking. This paper aims to fill this gap by investigating CC’s influence on CI and showcasing ongoing adaptation efforts to reduce these impacts.

This study reports on a study of bibliometric analyses and a set of case studies, which provides an overview of how CC influences infrastructure. The bibliometric analyses assessed the available literature and identified some current trends, which illustrate the diversity of recent works on the connections between CC and infrastructure, especially in industrialised countries, which were the main focus of the investigation. This study addresses a research gap in the literature by examining the impact of CC on CI, particularly in developing countries, while also highlighting ongoing CI projects and adaptation measures aimed at mitigating the effects of CC. This is important, since the analysis showed that the main research interests are focused on infrastructure related to water, either as a resource or as a problem within coastal infrastructures and human settlements.

The study emphasises the various climate and socioeconomic aspects of infrastructure systems and their features. It outlines the various possible damages from climate influences, especially extreme events. It focuses on water, human settlements, CI, coastal transport, crop systems, and energy.

Important medium- and long-term adaptation projects for CI carried out in various sectors and countries on all continents were identified among the lessons learned from the case studies. The importance of adapting CI lies in the fact that their lack of adaptation can lead to considerable damage due to the effects of CC, with interruptions that can be catastrophic for the population. Furthermore, multistakeholder engagement processes will also be essential in driving climate-resilient infrastructure pathways, as CIs mainly impact citizens and communities that could participate locally in designing adaptation plans.

The practical implications of this paper are two-fold. Firstly, it has outlined the many advantages of ensuring that measures are implemented to reduce the exposure of infrastructures to climate influences. Costs to owners and operators of infrastructure may be much higher if they fail to consider CC issues when the infrastructures are designed, built, and maintained. Secondly, the paper shows that the main research interests are focused on infrastructure related to water, either as a resource or as a problem within coastal infrastructures and human settlements.

This study has some limitations. The first is that the bibliometric analysis emphasised the literature in developed countries and needed to examine studies in other contexts, such as those focusing on developing nations. However, the case studies covered the five continents. Secondly, the bibliometric analysis needed more extensive to cover all infrastructure publications and focus on those more closely associated with climate influences.

Despite these constraints, the paper provides a welcome addition to the literature since it sheds some of the issues that permeate the impacts of CC on infrastructure and has addressed some of the information gaps seen in areas such as energy, which is critical to most countries. Greater awareness of the extent to which CC may threaten infrastructure may be an element that catalyses action towards increased protection of these facilities in response to changing climate conditions. Regarding future trends, adequate policies must be implemented to assist the various stakeholders, especially infrastructure owners, to take advantage of climate science better to guide their efforts to protect them. Also, further studies investigating how different methods could be deployed to protect infrastructure are needed to identify appropriate measures to replicate their use worldwide.

There are some lessons to learned, based on the experiences gathered during the research which led to this paper. The first one is that there is an urgent need for more efforts to better understand and manage the manifold impacts of CC on infra-structure. This is so, due to the many negative implications that damages to infra-structure can cause to the economy of a country, in particular developing ones. The second lesson learned is related to the fact that, whereas an emphasis is often given to impacts to energy and transport infra-structure, the importance of water-related ones should not be overlooked. Furthermore, it needs to be acknowledged that a proper monitoring of infrastructure is needed, to timely identify and potential problems, before the occur. Finally, there is a perceived need for broader awareness of the importance of paying a greater attention to CC, in the design and planning of future infrastructure projects, so these may be in a better position to cope with the pressures posed by changing climatic conditions.

In conclusion, this study underscores the urgent need for proactive measures to safeguard infrastructure from the impacts of CC. The findings emphasize the importance of integrating climate considerations into infrastructure planning, design, and maintenance processes. Failure to do so not only jeopardizes the resilience of infrastructure but also exposes owners and operators to increased costs and risks. Multistakeholder engagement and international cooperation are crucial in fostering climate-resilient infrastructure pathways, ensuring the well-being of communities worldwide. Moving forward, policymakers must prioritize investment in adaptation measures and greenhouse gas mitigation strategies to protect CI and enhance global climate resilience. Finally, to protect infrastructure from CC impacts, governments must invest in measures such as flood control, early warning systems, and improved building codes. Additionally, they need to work to reduce greenhouse gas emissions more actively, which are the primary cause of CC.

### Supplementary Information

Supplementary Information.

## Data Availability

The dataset generated and/or analysed during the current study are available in the Supplementary Data file.
